# Prognostic factors influencing the survival of patients with carcinoma of the gastric cardia receiving preoperative interventional embolization chemotherapy

**DOI:** 10.18632/oncotarget.18571

**Published:** 2017-06-19

**Authors:** Hai-Li Cao, Shu-Qiang Jin, Kai-Bing Wang, Bin Bai

**Affiliations:** ^1^ Department of Interventional Radiology, The Second Affiliated Hospital of Harbin Medical University, Harbin, 150086, China

**Keywords:** prognostic factors, carcinoma of gastric cardia, preoperative interventional embolization chemotherapy, retrospective analysis

## Abstract

The purpose of this study was to analyze the characteristics of patients with gastric cardia cancer (GCC) to identify the main factors the influence the survival rate after interventional embolization chemotherapy (IEC). One hundred and fifty-six patients with advanced GCC were treated with IEC via the left gastric artery. Survival time was defined as from the date of diagnosis until death or the end of this study in June 2015. The median survival time was 15 months (range 3 to 29 months). The Cox proportional hazard model found that patients’ age (*p* < 0.001), sex (*p* = 0.039), weight loss more than 10% in the prior 3 months (*p* = 0.014), body mass index (BMI) (*p* = 0.047), and hematocrit value less than 37% (*p* < 0.001) were correlated with mortality after removal of cases of poorly differentiated carcinoma and undifferentiated carcinoma from the analysis. Kaplan-Meier curves of survival according to patients’ age showed significant differences by the log-rank test (*p* = 0.0015). The median survival time was 17 months among patients of aged < 50 years. In conclusion, BMI, weight loss > 10% in the prior 3 months, albumin, and hematocrit were prognostic indicators for patients with advanced GCC, and patients younger than 50 years have a higher survival rate after IEC.

## INTRODUCTION

Carcinoma of the gastric cardia is a malignant tumor that occurs in the cardia and stomach and is a serious threat to human health. The etiology of gastric cardia cancer (GCC) is unclear, and may be related to dietary factors, environmental factors, genetic factors, and *Helicobacter pylori* infection [1–3]. Early GCC is generally asymptomatic and has the characteristics of high invasiveness and extensive metastasis. The 5-year survival rate is only around 20% postoperatively [4–8].

This soft tissue carcinoma forms at the bottom of the stomach and a continuous blood supply is necessary to support tumor invasion and growth. The major blood supply to this area is from the left gastric artery. Interventions to disrupt blood flow to carcinomas are an important technique in carcinoma treatment because they are minimally invasive and highly effective. Zhang et al. retrospectively analyzed 182 advanced GCC patients who were treated with selective left gastric artery and abdominal aorta interventional chemotherapy. Their results showed that all the patients with clinical symptoms improved after 1 month, and in 142 cases (78.02%), the tumor reduced in size [9]. Zhang et al. also observed the therapeutic effect of bottom left gastric artery interventional chemotherapy for 34 GCC patients. They concluded that interventional chemotherapy improves the clinical symptoms quickly with minimally invasiveness and the possibility of repeatability [10]. Therefore, arterial embolism chemotherapy and embolization can cause tumor necrosis and a reduction in tumor size, and may be able to prolong the survival time of patients with advanced GCC.

Iodized oil emulsion or gelatin sponge embolism of the left gastric artery can block the blood supply and cause tumor necrosis [11, 12]. Furthermore, interventional infusion of chemotherapeutic agents directly into the left gastric artery can have several advantages: first, the drug concentration in the tumor region is much higher compared with systemic intravenous infusion; second, it avoids metabolism or plasma protein binding of the chemotherapy drugs before they come into contact with the tumor cells; third, the drug concentration in other organs is much lower compared to that in intravenous chemotherapy. Therefore, interventional embolization chemotherapy (IEC) has a strong anti-cancer effect and can significantly reduce toxicity in comparison with systemic chemotherapy [13, 14].

Cardia cancer patients can be treated by IEC 7–14 days before radical resection, and it has the effect of causing tumor necrosis, reducing the tumor volume, reducing surgical bleeding, and shortening the operative time. Thus, IEC can not only improve tumor control, but can also improve the surgical resection rate, prevent iatrogenic diffusion, create the opportunity for surgery, and in theory prolong the patients’ survival time.

However, the 5-year survival rate has not improved in response to improvements in surgical techniques and preoperative treatment. The treatment outcome might be affected by different patients’ characteristics and pathological types. Although IEC is often applied before surgery, its effect on the patients’ outcome is still not clear. In this retrospective study, we analyzed the characteristics of patients to identify the main factors influencing the survival of patients.

## RESULTS

### Clinical characteristics of patients

In the present study, we enrolled 156 patients with GCC who received preoperative IEC. The median survival time was 15 months (range 3 to 29 months). Seven patients were alive at the end of this study. The included patients’ age was 56.4 ± 9.7 and men accounted for 66.7% of participants. Among all patients, patients with weight loss greater than 10% in the prior 3 months accounted for 86.5%; without autonomous activity, 87.8%; hypertension, 30.1%; diabetes, 21.2%; albumin less than 35 g/L, 78.2%; hematocrit value less than 37%, 49.4%. Patients who received preoperative systemic chemotherapy accounted for 28.8%, and 17.9% patients received preoperative radiotherapy. The number of IEC events was 1, 2, 3, 4, 5, 6, and 7 in 15, 24, 25, 39, 30, 19, and 4 patients, respectively. During the follow-up, 26.3% patients were found to have distant metastasis to other organs. There were 19 patients with undifferentiated carcinoma and 16 patients with poorly differentiated carcinoma (Table 1).

**Table 1:** Patient characteristics

Factors	Total	Group 1	Group 2	Group 3	*P* value
Age (year)	56.4 ± 9.7	44.0 ± 3.4	55.0 ± 2.6	67.0 ± 5.3	-
Gender					
Male	104 (66.7%)	30 (76.9%)	38 (60.3%)	36 (66.7%)	0.224
Female	52 (33.3%)	9 (23.1%)	25 (39.7%)	18 (33.3%)	
Weight loss ≥ 10%					
Yes	135 (86.5%)	31 (79.5%)	55 (87.5%)	49 (90.5%)	0.284
No	21 (13.5%)	8 (20.5%)	8 (12.5%)	5 (9.5%)	
Independent activity					
Yes	19 (12.2%)	38 (97.4%)	57 (90.5%)	42 (77.8%)	0.012
no	137 (87.8)	1 (2.6%)	6 (9.5%)	12 (22.2%)	
Hypertension					
Yes	47 (30.1%)	7 (17.9%)	14 (22.2%)	26 (48.1%)	0.002
no	100 (69.9%)	32 (82.1%)	49 (77.8%)	28 (51.9%)	
Diabetes mellitus					
Yes	33 (21.2%)	7 (17.9%)	15 (23.8%)	11 (20.4%)	0.769
No	123 (78.8%)	32 (82.1%)	48 (76.2%)	43 (79.6%)	
Smoking status					
Yes	59 (37.8%)	16 (41.0%)	21 (33.3%)	22 (40.7%)	0.636
No	97 (62.2%)	23 (59.0%)	42 (66.7%)	32 (59.3%)	
Alcohol drinking					
Yes	63 (40.4%)	20 (51.3%)	26 (41.3%)	17 (31.5%)	0.156
No	93 (59.6%)	19 (48.7%)	37 (58.7%)	37 (68.5%)	
History of chronic obstructive pulmonary disease					
Yes	54 (34.6%)	6 (15.4%)	25 (39.7%)	23 (42.6%)	0.014
No	102 (65.4%)	33 (84.6%)	38 (60.3%)	31 (57.4%)	
History of hepatitis					
Yes	37 (23.7%)	8 (20.5%)	15 (23.8%)	14 (25.9%)	0.832
no	119 (76.3%)	31 (79.5%)	48 (76.2%)	40 (74.1%)	
History of cardiovascular disease					
Yes	51 (32.7%)	4 (10.3%)	22 (34.9%)	25 (46.3%)	0.001
No	105 (67.3%)	35 (89.7%)	41 (65.1%)	29 (53.7%)	
Albumin (< 35 g/L)					
Yes	122 (78.2%)	28 (71.8%)	49 (77.8%)	45 (83.3%)	0.411
No	34 (21.8%)	11 (28.2%)	14 (22.2%)	9 (16.7%)	
Hematocrit value (< 37%)					
Yes	77 (49.4%)	20 (51.3%)	26 (41.3%)	31 (57.4%)	0.212
No	79 (50.6%)	19 (48.7%)	37 (58.7%)	23 (42.6%)	
Preoperative chemotherapy					
Yes	45 (28.8%)	17 (43.6%)	18 (28.6%)	10 (18.5%)	0.031
No	111 (71.2%)	22 (56.4%)	45 (71.4%)	44 (81.5%)	
Preoperative radiotherapy					
Yes	28 (17.9%)	11 (28.2%)	10 (15.9%)	7 (13.0%)	0.144
No	128 (82.1%)	28 (71.8%)	53 (84.1%)	47 (87.0%)	
Surgery time (> 30 min)					
Yes	72 (46.2%)	13 (33.3%)	33 (52.4%)	26 (48.1%)	0.161
No	84 (53.8%)	26 (66.7%)	30 (47.6%)	28 (51.9%)	
Duration of intermittent fever (days)					
1	13 (8.3%)	3 (7.7%)	10 (15.9%)	0 (0.0%)	–
2	30 (19.2%)	13 (33.3%)	11 (17.5%)	6 (11.1%)	
3	64 (41.0%)	15 (38.5%)	23 (36.5%)	26 (48.1%)	
4	36 (23.1%)	6 (15.4%)	14 (22.2%)	16 (29.6%)	
5	13 (8.3%)	2 (5.1%)	5 (7.9%)	6 (11.1%)	
Duration of intermittent vomiting (days)					
1	29 (18.6%)	8 (20.5%)	17 (27.0%)	4 (7.4%)	–
2	57 (36.5%)	20 (51.3%)	17 (27.0%)	20 (37.0%)	
3	60 (38.5%)	10 (25.6%)	24 (38.1%)	26 (48.1%)	
4	10 (6.4%)	1 (2.6%)	5 (7.9%)	4 (7.4%)	
Duration of intermittent upper abdominal pain (days)					
2	6 (3.8%)	5 (12.8%)	1 (1.6%)	0 (0.0%)	–
3	41 (26.3%)	15 (38.5%)	19 (30.2%)	7 (13.0%)	
4	56 (35.9%)	16 (41.0%)	23 (36.5%)	17 (31.5%)	
5	39 (25.0%)	3 (7.7%)	15 (23.8%)	21 (38.9%)	
6	12 (7.7%)	0 (0.0%)	5 (7.9%)	7 (13.0%)	
7	2 (1.3%)	0 (0.0%)	0 (0.0%)	2 (3.7%)	
Postoperative eat to alleviate time (days)					
4	6 (3.8%)	2 (5.1%)	3 (4.8%)	1 (1.9%)	–
5	30 (19.2%)	10 (25.6%)	17 (27.0%)	3 (5.6%)	
6	49 (31.4%)	16 (41.0%)	13 (20.6%)	20 (37.0%)	
7	54 (34.6%)	10 (25.6%)	23 (36.5%)	21 (38.9%)	
8	17 (10.9%)	1 (2.6%)	7 (11.1%)	9 (16.7%)	
Organ metastasis					
Yes	41 (26.3%)	11 (28.2%)	19 (30.2%)	11 (20.4%)	0.464
No	115 (73.3%)	28 (71.8%)	44 (69.8%)	43 (79.6%)	
Histotype					
Poorly differentiated carcinoma	16 (10.3%)	3 (7.7%)	5 (7.9%)	8 (14.8%)	–
Tubular adenocarcinoma	40 (25.6%)	5 (12.8%)	13 (20.6%)	22 (40.7%)	
Polypoid adenocarcinoma	38 (22.4%)	16 (41.0%)	18 (28.6%)	4 (7.4%)	
Undifferentiated carcinoma	19 (12.2%)	2 (5.1%)	7 (11.1%)	10 (18.5%)	
Signet-ring cell carcinoma	15 (9.6%)	1 (2.6%)	9 (14.3%)	5 (9.3%)	
Mucinous adenocarcinoma	28 (17.9%)	12 (30.8%)	11 (17.5%)	5 (9.3%)	
Survival time (months)	15.0 (2.0–24.0)	18.0 (5.0–24.0)	16.0 (4.0–24.0)	9.0 (3.0–24.0)	< 0.001

The χ^2^ test was used to evaluate differences across the three age groups of patients. The number of patients with autonomic activity (*p* = 0.012), hypertension (*p* = 0.002), history of COPD (*p* = 0.014), history of cardiovascular disease (*p* = 0.001), and received preoperative chemotherapy (*p* = 0.031) were significantly different among the groups. For mortality, 143 patients died within 2 years after surgery and the overall survival rate was 8.3%. There was no statistical difference across age groups.

### Cox multivariate analysis

In the Cox proportional hazard model, we found that the patients’ age (HR: 1.07, 95% confidence interval [CI]: 1.044–1.096, *p <* 0.001), sex (HR: 0.615, 95% CI: 0.386–0.98, *p* = 0.041), weight loss more than 10% in the prior 3 months (HR: 2.3, 95% CI: 1.126–4.698, *p* = 0.022), albumin less than 35 g/L (HR: 1.907, 95% CI: 1.058–3.438, *p* = 0.032), and hematocrit value less than 37% (HR: 5.353, 95% CI: 3.419–8.380, *p <* 0.001) were correlated with the risk of death of cardia cancer patients after IEC (Table 2).

**Table 2:** Cox proportional hazard model analysis of the risk of death in all cardia cancer patients

Variable	Haz. Ratio	Std. Err.	z	*P* value	[95% Conf.	Interval]
Age	1.070	0.013	5.45	< 0.001	1.044	1.096
Sex	0.615	0.146	–2.04	0.041	0.386	0.980
Weight loss > 10% in 3 months	2.300	0.838	2.29	0.022	1.126	4.698
BMI	0.767	0.118	–1.73	0.084	0.568	1.036
Autonomous activity	0.882	0.247	–0.45	0.653	0.510	1.526
Hypertension	1.319	0.289	1.26	0.206	0.859	2.026
Diabetes	0.762	0.185	–1.12	0.262	0.474	1.225
Smoker	0.955	0.234	–0.19	0.851	0.591	1.544
Drink wine	1.089	0.224	0.42	0.678	0.728	1.629
History of chronic obstructive pulmonary disease	0.994	0.234	–0.03	0.979	0.627	1.575
History of hepatitis	1.427	0.334	1.52	0.129	0.902	2.257
History of cardiovascular disease	1.108	0.246	0.46	0.645	0.716	1.713
Albumin less than 35 g/L	1.907	0.573	2.15	0.032	1.058	3.438
Hematocrit value less than 37%	5.353	1.224	7.33	< 0.001	3.419	8.380

For pathological types and TNM stage, metastasis was only found in patients with poorly differentiated carcinoma (6/16) and undifferentiated carcinoma (17/19) (Table 3). After removal of poorly differentiated carcinoma and undifferentiated carcinoma patients from the analysis, the Cox proportional hazard model showed that patients’ age (HR: 1.089, 95% CI: 1.056–1.122, *p <* 0.001), sex (HR: 0.546, 95% CI: 0.308–0.969, *p* = 0.039), weight loss more than 10% in the prior 3 months (HR: 2.632, 95% CI: 1.215–5.700, *p* = 0.014), BMI (HR: 0.693, 95% CI: 0.483–0.995, *p* = 0.047), and hematocrit value less than 37% (HR: 5.715, 95% CI: 3.354–9.738, *p <* 0.001) were correlated with the mortality of cardia cancer patients (Table 4).

**Table 3:** Characterization of pathological types and TNM stage in all cardia cancer patients

		Pathological types						
	Group	Poor differentiated carcinoma	Tubular adenocarcinoma	Papillary adenocarcinoma	Undifferentiated carcinoma	Signet-ring cell carcinoma	Mucinous adenocarcinoma	Total
Metastasis	No	10	40	38	2	15	28	133
	Yes	6	0	0	17	0	0	23
	Total	16	40	38	19	15	28	156

**Table 4:** Cox proportional hazard model analysis of risk of death in relatively well pathological type patients

Variable	Haz. Ratio	Std. Err.	z	*P* value	[95% Conf.	Interval]
Age	1.089	0.017	5.49	< 0.001	1.056	1.122
Sex	0.546	0.160	–2.07	0.039	0.308	0.969
Weight loss > 10% in 3 months	2.632	1.038	2.45	0.014	1.215	5.700
BMI	0.693	0.128	–1.99	0.047	0.483	0.995
Autonomous activity	1.073	0.380	0.2	0.842	0.537	2.147
Hypertension	0.871	0.243	–0.5	0.620	0.504	1.505
Diabetes	0.744	0.213	–1.03	0.302	0.425	1.303
Smoker	0.733	0.213	–1.07	0.285	0.415	1.295
Drink wine	1.033	0.260	0.13	0.898	0.630	1.692
History of chronic obstructive pulmonary disease	0.767	0.230	–0.89	0.376	0.426	1.380
History of hepatitis	1.212	0.367	0.64	0.525	0.670	2.195
History of cardiovascular disease	1.499	0.410	1.48	0.139	0.877	2.564
Albumin less than 35 g/L	1.700	0.575	1.57	0.117	0.876	3.300
Hematocrit value less than 37%,	5.715	1.554	6.41	< 0.001	3.354	9.738

### Survival analysis

**Figure 1:**
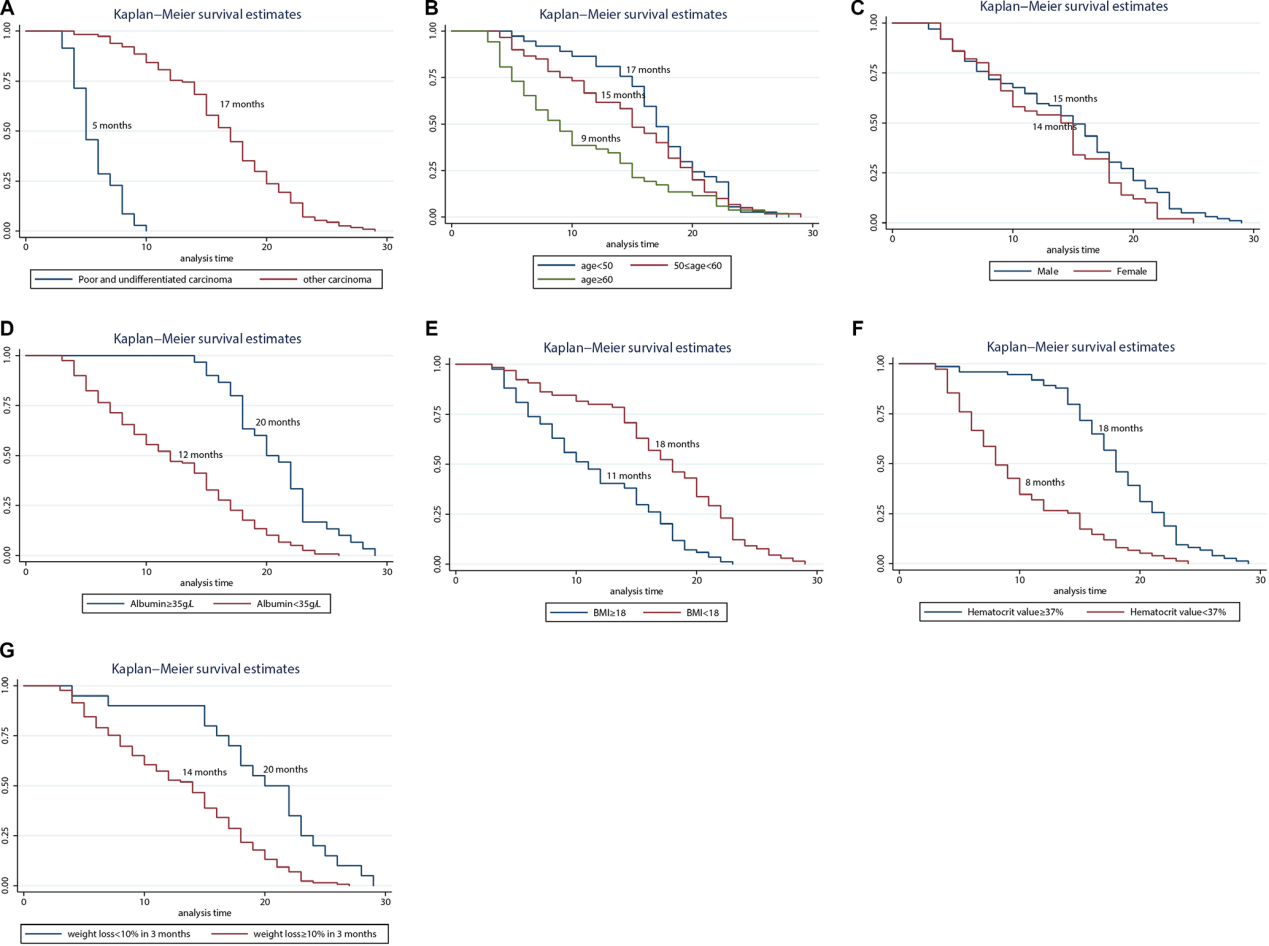
Kaplan-Meier curves of overall survival according to pathological type (**A**) age (**B**) sex (**C**) albumin (**D**) BMI (**E**) hematocrit value (**F**) and weight loss > 10% in 3 months (**G**).

Figure 1A shows the Kaplan-Meier curves of overall survival according to pathological type. The log-rank test showed a significant difference between patients with different types (*p <* 0.001). The median survival time of cases with poorly differentiated and undifferentiated carcinoma was 5 months, but for the other pathological types it was 17 months.

Figure 1B shows the Kaplan-Meier curves of survival according to patients’ age with significant differences by the log-rank test (*p* = 0.0015). The median survival time was 17 months for patients aged < 50 years age, 15 months for patients aged 50 to 60 years, and 9 months for patients aged > 60 years. Thus, after treatment with IEC, younger patients (aged < 60 years) had a longer survival period than the general end-stage cardia cancer survival period (around 1 year in our hospital’s experience). However, the median survival time between male and female patients did not show significant differences (0.0791): 15 months for men and 14 months for women (Figure 1C).

In the present study, the median survival time of patients with different albumin concentrations was significantly different (*p* = 0.001). The median survival time of patients with albumin less than 35 g/L was 12 months, and for patients with albumin greater than 35 g/L, it was 20 months (Figure 1D). BMI was also significantly correlated with patients’ survival time (*p <* 0.001). However, the median survival time of patients with BMI < 18 kg/m^2^ was 11 months compared with 18 months in patients with BMI ≥ 18 kg/m^2^ (Figure 1E). Similar to the COX analysis, the median survival time of patients with a hematocrit value < 37% was 8 months vs. 18 months for patients with > 37%, and the difference was significant (*p* < 0.001) (Figure 1F). Rapid weight loss may reflect rapid tumor growth. The median survival time was 14 months in patients with weight loss > 10% in the prior 3 months, and it was significantly different from the 20-month survival time in patients without rapid weight loss (*p <* 0.001) (Figure 1G).

## DISCUSSION

In this retrospective study, we enrolled 156 GCC patients who received preoperative IEC. During the follow-up, 149 patients died, a fatality rate of 95.5%, and the median survival time was 15 months. Cox proportional hazard model results identified factors correlated with mortality, including age, sex, weight loss, BMI, albumin < 35 g/L, and hematocrit value < 37%. In survival analysis, the survival period was significantly different in patients with different pathological types. In addition, patients’ age, albumin level, BMI, hematocrit value, and weight loss were also important factors correlated with patients’ survival time.

The blood supply to the gastric fundus and cardia mainly originates from the left gastric artery, which is the main blood vessel utilized in interventional therapy. Chemotherapy and embolization can block the tumor’s blood supply, increase the local concentration of antineoplastic drugs, and reduce the probability of gastric ulcer and necrosis [11, 12]. The patients can also be treated with a mixture of oxaliplatin and lipiodol, which can release the drug slowly, causing a continuous tumor killing effect. Preoperative IEC is relatively effective in controlling local disease. Prior studies have shown that preoperative interventional chemotherapy can obviously improve the GCC surgery success rate and that the 1-year survival rate was 81.2% [11]. However, another study reported that the 1-year survival rate of GCC patients treated with interventional chemotherapy and embolism with radiotherapy was only 52.3% [12]. All of the patients included in the current study were in an advanced stage; all patients had lymph node metastases, and 23 patients had distant metastases, and therefore the survival time of the study population was very short. The 1-year survival rate was 57%, and the median survival time was 15 months.

Age is thought to be a factor that affects the survival of cancer patients. One study found that increased age and post-operative respiratory insufficiency were correlated with a shorter survival [15]. This study also found that age was correlated with the survival rate, but there is still a need for future studies to determine as to whether there is a correlation between respiratory function and survival rate. In other studies, an age > 65 years was an independent risk factor for the mortality of proximal gastric cancer patients, similar to our results [16]. In the comparative results of cardia cancer patients treat by IEC in our study, the age < 50 group had a significantly longer survival than the 50–60 age group, and the 50–60 age group had a significantly longer survival than the age > 60 group, who only had a median 18 months survival time. However, the median survival time of the three groups was not significantly different. The main reasons may be the difference in biological responses of tumors to chemotherapy and individual biological differences, and therefore, the effect of age and chemotherapy is weakened.

In our analysis, the number of patients with autonomic activity, hypertension, history of COPD, history of cardiovascular disease, and received preoperative chemotherapy were significantly different among the three age groups. This indicates the different age groups have different characteristics of disease that may affect survival time. The majority of patients enrolled in this study were men, and men had a higher risk of mortality. Other studies have shown that young men also have a higher prevalence of GCC [17]. However, few studies have reported an effect of sex on the survival of patients, which is in need of further research.

The body weight and BMI reflects the nutritional status of patients. Some studies found the BMI has a strong correlation with the prevalence of esophageal cancer and GCC; they also found the prevalence increased 1.11 per each BMI increase of 5 kg/m^2^. Interestingly, our study indicated mortality of patients with BMI > 18 kg/m^2^ is lower than patients with BMI < 18 kg/m^2^ and that maybe related to the higher calorie consumption in patients with BMI < 18 kg/m^2^. Therefore, improvements in the nutritional status of patients may be important for the survival of patients.

Low serum albumin also reflects the patients` protein consumption, which indicates an imbalance in the patients’ nutrition. Lien et al. found that low preoperative albumin levels were highly correlated with resectability and survival of adenocarcinoma and gastric cardia patients [18]. Our results also showed patients with abnormal serum albumin levels had shorter survival times than those with normal serum albumin levels. The hematocrit value refers to the percentage of erythrocyte volume in the whole blood, which is usually reduced after serious trauma and shock. Our research also found the patients with low hematocrit values had shorter survival times.

In addition, some researchers believe that the survival rate of cardia cancer is related to the expression of the RAS-association domain family and 2, 6 methylation [19]. Cardia cancer patients` survival time has also been reported to be related to lymph node metastasis [20]. The patients included in our study all had lymph node metastasis; that may be the reason for the higher mortality rate compared with other studies.

In studies of GCC, prevalence and surgical methods have been extensively researched, but studies of survival analysis are not as plentiful. Our survival analysis study researched advanced GCC patients with IEC, and found that age, sex, weight loss, BMI, albumin, and hematocrit value might be correlated with patients` survival time. This study will be helpful for prognostication of GCC patients undergoing IEC.

There were several limitations to our research. First, the long retrospective period can cause bias because of improvements in surgery and nursing. Second, the retrospective design of this study may reduce the reliability of the results. Third, there are additional potential risk factors that were not analyzed in our study.

According to our analysis of survival data of advanced GCC patients, we found that patients younger than 50 have a higher survival rate after IEC. Thus, this treatment approach has a relatively high clinical application value. We found that BMI, recent weight loss > 10%, albumin, and hematocrit levels were prognostic indicators. Furthermore, whether the survival rate could be improved by actively treating the above indicators is worthy of further research and analysis. However, further studies are still needed to verify the robustness of our results because of the heterogeneity of the treatments and our study’s retrospective design.

## MATERIALS AND METHODS

### Study population and clinical management

From January 2001 to January 2013, 156 patients with advanced GCC were treated with IEC in the left gastric artery (at the second affiliated hospital of Harbin Medical University). The inclusion criterion were as follows: > 18 years old; advanced carcinoma of gastric cardia confirmed by histologic diagnosis; receiving IEC; normal renal function that was defined as having a normal serum creatinine level; a white blood cell (WBC) count > 3.5 × 10^9^/liter and platelets > 100 × 10^9^/liter; complete surgical tumor resection; and having complete disease and follow-up records. The exclusion criteria were other severe systemic diseases; known chronic digestive system disease; history of other gastric surgery; or incomplete disease and follow-up records. This study did not limit inclusion by the tumor node metastasis (TNM) stage or pathological types of tumors.

In this retrospective study, the predefined observed indicators included age, sex, weight loss more than 10% in the prior 3 months, body mass index (BMI), autonomous activity, hypertension, diabetes, smoking, drinking alcohol, history of chronic obstructive pulmonary disease, history of hepatitis, history of cardiovascular disease, albumin less than 35 g/L, hematocrit value less than 37%, preoperative systemic chemotherapy, preoperative radiotherapy, surgery time longer than 30 minutes, duration of fever, duration of vomiting, duration of upper abdominal pain, postoperative time to oral feeding, distant metastasis on follow-up, pathological types of tumors, and survival time.

All patients agreed to participate after explanation of the risks and benefits, including possible complications of the operation and the possible need for additional surgery. Written informed consent was obtained from all patients, and the protocol was reviewed and approved by the Ethics Committee of the second affiliated hospital of Harbin Medical University.

### IEC procedures

**Figure 2:**
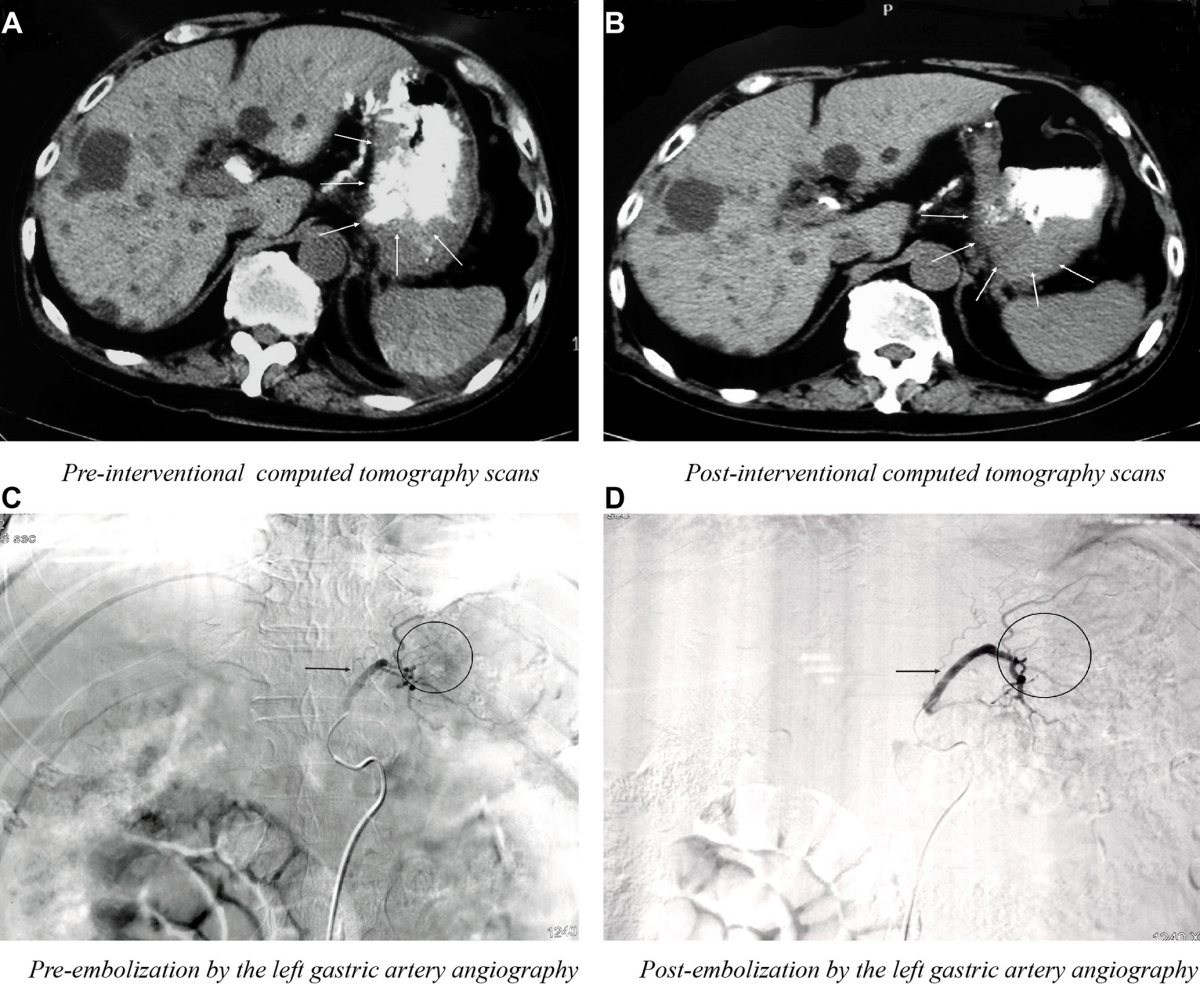
The results of pre-interventional (**A**) post-interventional (**B**) computed tomography scans, pre-embolization (**C**) and post-embolization (**D**) by the left gastric artery angiography.

After local anesthesia and disinfection, celiac trunk angiography was performed through the patient’s right femoral artery using a 4F Cobra catheter (Soft-Vu^®^, NY, U.S.). The catheter was entered into the left gastric artery super-selectively, and then we slowly perfused 40–60 ml floxuridine and epirubicin. Then, a lipiodol and oxaliplatin emulsion was used to block the vessel until the blood flow slowed down significantly and there was a disappearance of tumor staining. The results of pre-interventional, post-interventional computed tomography scans, pre-embolization and post-embolization by the left gastric artery angiography are presented in Figure 2.

### Follow-up

Post resection surgery, all patients’ vital signs were observed closely. Gastric mucosal protection, nutritional support, anti-tumor, and symptomatic treatment were supplied until the patient was stable. Additional treatment was implemented according to the patients’ tumor changes, and the number of interventional treatments was recorded. All patients were followed-up until death or the end of this study in June 2015.

### Statistical analysis

The qualitative data were expressed as frequency (percentage) and statistical significance was evaluated by use of the χ^2^ test and the Kruskal-Wallis test. Quantitative data were expressed as mean ± standard deviation, and we performed the group comparison using analysis of variance (ANOVA) if the data had a normal distribution. If not, the data were expressed as median and quartile, and we compared the groups using a nonparametric test. The observation time for the survival analysis was from the date of diagnosis until death or the end of this study in June 2015. A multivariate Cox proportional hazards model was used to estimate multivariate hazard ratios (HRs) for overall survival. Differences in Kaplan-Meier survival curves were tested using the log-rank test. Results with *p <* 0.05 were defined as significant.

### Ethical approval

The study was reviewed and approved by the Ethics Committee of the second affiliated hospital of Harbin Medical University.
